# What’s All the Chatter? A Mixed-Methods Analysis of Emergency Physicians’ Tweets

**DOI:** 10.5811/westjem.2019.10.44004

**Published:** 2019-12-09

**Authors:** Jeff Riddell, Alisha Brown, Lynne Robins, Rafae Nauman, Jeanette Yang, Joshua Jauregui

**Affiliations:** * University of Southern California Keck School of Medicine, Department of Emergency Medicine, Los Angeles, California; † University of Washington, Department of Emergency Medicine, Seattle, Washington; ‡ University of Washington, Department of Biomedical Informatics and Medical Education, Seattle, Washington; § University of Nevada, Las Vegas School of Medicine, Las Vegas, Nevada; ¶ University of Washington, Department of Surgery-Surgical Outcomes Research Center, Seattle, Washington

## Abstract

**Introduction:**

Twitter is growing in popularity and influence among emergency physicians (EP), with over 2200 self-identified EP users. As Twitter’s popularity has increased among EPs so too has its influence. While there has been debate about the value of Twitter as an effective educational delivery tool, little attention has been paid to the nature of the conversation occurring on Twitter. We aim to describe how influential EPs use Twitter by characterizing the language, purpose, frequencies, content, and degree of engagement of their tweets.

**Methods:**

We performed a mixed-methods analysis following a combined content analysis approach. We conducted qualitative and quantitative analyses of a sample of tweets from the 61 most influential EPs on Twitter. We present descriptive tweet characteristics and noteworthy themes.

**Results:**

We analyzed 1375 unique tweets from 57 unique users, representing 93% of the influential Twitter EPs. A majority of tweets (1104/1375, 80%) elicited some response in the form of retweets, likes, or replies, demonstrating community engagement. The qualitative analysis identified 15 distinct categories of tweets.

**Conclusion:**

Influential EPs on Twitter were engaged in largely medical conversations in which most messages generated some form of interaction. They shared resources and opinions while also building social rapport in a community of practice. This data can help EPs make informed decisions about social media engagement.

## INTRODUCTION

Twitter is a social media platform that allows individuals to communicate through short, 280-character messages that are accessible to the public. Twitter has grown in popularity and influence among emergency physicians (EP) with over 2200 self-identified users in 2013.^1^ More than a quarter of emergency medicine (EM) faculty now use Twitter.^2^ EPs use Twitter for both formal and informal reasons including discussing clinical cases, collaborating on and disseminating research, advocating for patients, participating in journal clubs, promoting educational messages from national conferences, and providing feedback to learners.^3^–^9^ Some have even suggested that Twitter has facilitated the formation of virtual communities of practice among its users.^10^,^11^

As Twitter’s popularity has increased among EPs so too has its educational influence.^12^ While there has been debate about the value of Twitter as an effective educational delivery tool, little attention has been paid to the nature of the conversation occurring on Twitter.^12^–^17^ As the clinical and academic interaction among EPs continues to grow on social media platforms, a more robust understanding of the characteristics of these interactions can help provide a framework for conscientious EPs to consider whether Twitter represents a platform for meaningful communication among a professional digital community of practice or simply a “insubstantive fragmented” stream of “doubtful significance.”^15^,^17^

This study addresses this gap by analyzing the messages of influential EPs on Twitter. We sought to describe the current state of Twitter usage among EPs by exploring the tweets of influential EPs. A deep exploration of the language, frequencies, domains, and degrees of engagement of their messages can provide a contextualized understanding of the real-life Twitter experience, allowing faculty and trainees to make mindful decisions about social media participation.

### Objective

The purpose of this study was to describe the nature of EPs’ communications on Twitter by characterizing the language, purpose, frequencies, content, and degree of engagement of their tweets.

## METHODS

### Study design

We performed a mixed-methods analysis following a combined content analysis approach. Because text is originally qualitative, and the quantification of text alone is insufficient for successful understanding of content,^18^ combined content analysis has been suggested to address the mixed nature of Twitter feed data in a single study.^19^

### Sample

We conducted qualitative and quantitative analyses of a sample of tweets from the 61 most influential EPs on Twitter, as defined in a previous study.^12^ We chose to include tweets from influential EPs because they disproportionately impact the spread of information and directly shape social media conversations.^20^, ^21^,^22^ As demonstrated in previous studies, analyzing influencers yields a broad description of Twitter activity without having to analyze all users.^23^ As such, the tweets of the most influential EPs were likely to provide a narrative that reflected the general conversation of EPs on Twitter.

### Data Source and Search Strategy

To avoid variation in tweet content due to world events, national professional conferences, and seasonal variation, we analyzed tweets from random days in 2015. Specific days were identified using a random date generator function in Microsoft Excel (Redmond, WA, 2016). Once these dates were identified, we used the Twitter Advanced Search (https://Twitter.com/search-advanced?lang=en) function to identify and download all tweets produced by the influential EPs on these random days. We stopped our analysis after 10 days of tweets when we reached theoretical sufficiency in the qualitative^15^,^17^,^24^ component. Previously published Twitter content analyses outside of EM have examined between 288 and 12,666 tweets.^25^,^26^,^27^,^28^

**Educational Research Capsule Summary**

What do we already know about this issue?

*Twitter is popular among emergency physicians*.

What was the research question?

What is the nature of influential emergency physicians’ communications on Twitter?

What was the major finding of the study?

*A majority of tweets elicited some engagement. The qualitative analysis identified 15 distinct categories of tweets*.

How does this improve population health?

*This data can help emergency physicians make informed decisions about social media engagement*.

We included all original tweets that appeared in the Twitter Advanced Search timeline of the influential EPs on the selected days. Our corpus included original tweets, replies, and modified re-tweets. It did not include any unmodified re-tweets (messages that pass along another user’s tweet to one’s followers without adding one’s own comment or opinion).

### Qualitative Component

We analyzed the content of the of tweets using a naturalistic inductive content analysis approach.^19^ Four authors (AB, JR, JY, and RN) initially read all tweets line-by-line through the first three days of tweets and met to develop and refine the initial coding categories in an inductive manner. We developed and clarified the coding categories in an iterative approach and identified tweets to serve as unambiguous examples, which allowed each relevant item from a single tweet to be placed into a category. All authors met to discuss and distinguish between descriptive and thematic categories. We used the languages of content analysis and conversation analysis as sensitizing frameworks to guide the a priori determination of descriptive categories for our qualitative analysis.^29^,^30^,^31^ Thematic categories were identified using an iterative approach to coding. The first day’s tweets were used exclusively for code development, and were excluded from further analysis.

After the initial code and categorical development, two team members (JY and RN) coded the remaining tweets. Any disagreements were brought to the coding team for resolution.

Our experiences, backgrounds, and assumptions influence our approaches to analysis, so we chose a coding team with diverse experience with EM Twitter.^32^ Three authors are EPs (AB, JJ, and JR). The lead author (JR) has extensive Twitter experience including daily use of the platform, and was positively predisposed toward Twitter. One author (AB) rarely uses Twitter, and brought a more neutral lens to the analysis. Two authors (JY and RN) were undergraduate students with no experience on Twitter, created accounts solely for the purpose of this study, and had minimal preconceptions about physicians on Twitter. One author (LR) has background training in anthropology, and extensive experience using qualitative research methods in health professions education.

To enhance the trustworthiness and credibility of our data analysis we employed memoing, reflexivity, triangulation of data among researchers, and the formation of an audit trail of the analytical process.

### Quantitative Component

For each individual tweet we recorded message-level data to better understand tweet engagement. We defined tweet engagement by the number of retweets, “likes” (when another user clicks a heart on the message, generally indicating some form of agreement), and replies (the number of responses to a tweet prior to the author re-entering the conversation). We also logged the use of hyperlinks, embedding of media (pictures or video), and the first three hashtags (a type of metadata tag that makes it possible for others to easily find messages with a specific theme or content) per message. We also recorded the number of times each of the qualitatively-derived categories were applied to a tweet. Descriptive statistics were used to analyze this data.

## RESULTS

We analyzed 1375 unique tweets from 57 unique users, representing 93% of the influential Twitter EPs. Four (7%) influential users did not record any tweets on the sample of days analyzed. Quantitatively, a majority of tweets (1104/1375, 80%) had some engagement in the form of retweets, likes, or replies. The mean number of times a tweet was retweeted by another user was 2.1 (standard deviation [SD] 7.24), liked was 3.4 (SD 9.4), and replied to (messages from others before the original tweet author re-entered the conversation) was 0.8 (SD 1.4).

There were 448 hashtags used, occurring in 337/1375 (25%) tweets. The most common hashtags used are displayed in Table 1. #smaccUS and #FOAMed were the most common, occurring in 6.5% (90/1375) and 6.4% (88/1375) of tweets, respectively.

The qualitative analysis identified 15 distinct descriptive categories and eight thematic categories of tweets. Descriptive categories of tweet characteristics are presented in Table 2. Messages were split evenly between initiations of new conversation and replies to other tweets. While most tweets were statements, 22% were either questions or answers. Most were related professionally to the broad domain of medical practice, while fewer were social in nature. Interestingly, 13% of tweets served to change the domain of the conversation, blending the medical and social. The valence of most tweets was neutral, with only 3% expressing a negative tone, attitude, or feeling.

Noteworthy thematic categories with exemplary tweets are presented in Table 3. Over a quarter of tweets (375/1375, 27%) contained a summary of a resource, generally with a hyperlink to a blog post, journal article, podcast episode, or third-party website containing clinical information. Nearly a quarter of tweets (336/1375, 24%) contained illuminating statements that provided new perspective to move a conversation forward. These messages often added a different interpretation of clinical practice from one’s own experience. Rapport building (252/1375, 18%) and humor (165/1375, 12%) were also prevalent. Self-promotion and advertisements were less common, occurring in less than 5% of tweets. Although also rare, some tweets (31/1375, 2%) contained reflections on character, actions, professional practice, and relationships.

## DISCUSSION

Our results provide a contextualized understanding of the real-life EM Twitter experience, enabling EPs to make mindful decisions about social media participation. While the conversation skewed to medical topics, there was a significant social component to the interactions we analyzed. Humor, networking strategies, and rapport-building messages were common, revealing a human side to the EM Twitter conversation. Although not surprising given the “social” nature of social media and physicians’ desires to connect,^33^ the blend of personal and medical tweets highlights the ways in which social media tangles with traditional notions of friendships with colleagues outside of work.^34^

Influential EPs on Twitter also demonstrated a shared domain of interest (EM) and helped each other by sharing information and building relationships. These characteristics are consistent with traditional notions of a community of practice (CoOP).^35^ Within CoOPs, interpersonal professional connections have traditionally been limited by geographic spread, organizational hierarchies, and institutional siloing.^36^ Twitter may offer a new opportunity to weave a more accessible human element into the fabric of professional conversations, fostering the development of the relationships and networks that are important to organizational development, engagement, and vitality.^37,38^ The emergence of a Twitter CoOP among EM and critical care may enable relational and professional communication among colleagues who might not otherwise connect due to structural, political, or geographic barriers.^11^ While Twitter can break down traditional hierarchical structures and barriers to collaboration, education, and innovation, new challenges emerge that require “reconciliation.”^39^

Wenger-Trayner’s metaphor of “landscapes of practice” highlights the ways in which professionals negotiate their identities among many different CoOPs.^40^ In an increasingly complex “landscape” that involves several local (administration, clinical practice, teaching, etc), and now virtual (Twitter), communities, our findings support the notion that EPs are working to negotiate a productive identity with respect to the various CoOPs that constitute this landscape. Through self-promotion and networking messages, users were moving between and bridging CoOPs to connect their scholarly work (local or national research CoOPs) with their social media colleagues (Twitter CoOP). The use of hashtags like #smaccUS and #EMconf demonstrate how users blur the boundaries between traditional communities built around contemporaneous co-located educational conferences and their asynchronous virtual community.

The influential EPs we studied were innovators who formed the EP Twitter community based on egalitarian principles,^41^ and our data elaborate on their willingness to share resources and connect with the community. However, as previous professional boundaries blur, it is possible that new professional silos will emerge in their place. Could EM Twitter become the hierarchy from which a new group of “outsiders” could feel ostracized? Are there non-influential outsiders within EM Twitter that feel like the community is not theirs?

Future work might explore the perceived value of Twitter to the individual EPs who use the platform. While humor, sharing resources, networking, retweets, likes, and replies may appear on the surface to represent connection to the EM community, we did not explore whether Twitter users truly experienced this sense of connection. A recent study demonstrated that young adults with high social media use feel more socially isolated than their counterparts with lower social media use.^42^ While influential EPs may appear to be connecting on Twitter, they may actually feel socially isolated. Likewise, those not actively engaged in the Twitter conversation may feel like outsiders peering in on a community to which they are not connected. This topic of perceived vs lived experiences of connection is ripe for future inquiry.

Our data suggests that people are engaging in conversation and interacting by exchanging resources, creating new contacts, sharing ideas, thoughts, and reflections. While we see this broadly as a positive trend, it may be dangerous if, as has been reported, half of medical tweets from professional accounts are inaccurate.^16^ We did not evaluate the scientific accuracy of any tweets, nor did we examine the content of tweets for issues of professionalism or violations of privacy. These important issues deserve further exploration.

## LIMITATIONS

We analyzed English-language content only and findings may not generalize to the global medical community. We chose to analyze tweets from random days, allowing for the possibility that we may have missed significant and/or meaningful events in the EM community that could have changed the nature of the conversation and thus our conclusions. While we analyzed influential EPs due to the way they disproportionately impact the spread of information and directly shape social media conversations, our analysis may not reflect the lived experience of all EPs on Twitter. Further, the subjects we studied were deemed most influential from data analyzed in 2015. As EM Twitter rapidly evolves, those driving the discourse today may be significantly different from the influencers of three years ago. In particular, the representation of females on the list of influential Twitter users that we used was likely not representative of the EM social media community as a whole.

## CONCLUSION

Influential emergency physicians on Twitter were engaged in largely medical conversations in which most messages generated some form of interaction. They shared resources and opinions while also building social rapport in a community of practice. This data can help emergency physicians make informed decisions about social media engagement.

## Figures and Tables

**Table 1:** Most commonly used hashtags among tweets of influential emergency physicians.

Hashtag	Incidence (n=1375)
#smaccUS	90 (6.5%)
#FOAMed	88 (6.4%)
#EMconf	12 (0.8%)
#MEMC15	12 (0.8%)
#Read	10 (0.7%)
#smaccDUB	10 (0.7%)

**Table 2:** Descriptive categories of tweet characteristics of influential emergency physicians.

Tweet characteristic*	Definition	N (of 1375)	%
Position of message			
Initiation	The first tweet in a conversation, including retweets (RT) in which words are inserted prior to the RT message. Also includes modified tweets	673	49%
Reply	A response to any message from another user.	702	51%
Type of message**			
Question	A tweet worded or expressed so as to elicit information from other users. Not every tweet with a question mark fits here. For example, if a linking article has a question mark in the title, this does not count as a question on its own.	140	10%
Statement	Making a declarative initiation or reply, including rhetorical questions.	1117	81%
Answer	A reply to another user’s question.	166	12%
Domain***			
Medical	Pertaining to medicine or the broad domain of professional practice as a physician (this is NOT about the words used in the tweet, it is about the context of the conversation).	964	70%
Social	Unrelated to medicine - may be personal, cultural, political.	411	30%
Blend	A reply (not initiation) tweet that signals a change in the tone of the conversation between medical and social (can blend in either direction).	176	13%
Evaluate			
Yes	User adds his/her own judgment or opinion on the significance, worth, or quality of something. For example: “totally agree - just don’t have much luck admitting elsewhere due to rapid response parameters” was considered evaluative.	636	46%
No	User does not add his/her own judgment or opinion on the significance, worth, or quality of something.	739	54%
Valence			
Positive	Positive intrinsic feeling, emotional tone, or attitude expressed.	323	23%
Negative	Negative intrinsic feeling, emotional tone, or attitude expressed.	47	3%
Neutral	Default to neutral if not clearly positive or negative.	1005	73%

* Categories of tweet characteristics were defined *a priori* but derived qualitatively using the methodology referenced above.

** Several tweets were dual coded as both answering a question and asking another. Or making a statement and asking a question.

*** Each tweet was coded as either medical or social. If there was a change in the tone over the course of a conversation, it could receive an additional code as a “blend.” In blended tweets, the initial domain was coded.

**Table 3:** Thematic categories of tweets of influential emergency physicians.

Theme	Definition	Exemplary tweet	N (of 1375)	%
Resource summary	A mostly sterile accounting of the main points of something – including the title of a linked to resource or the summary of a case.	The problem with calf clots? Everyone handles them differently...and @emergencypdx explains why http://blog.ercast.org/the-problem-with-calf-clots/ … #FOAMed	375	27%
Rapport building	Explicitly pursuing relational connection, especially harmonious or sympathetic relation.	@JohnPurakal @mksheehy @UICBrownCoat Really great idea and stellar start. Can't wait for the next video! Keep up the good work.	252	18%
Illumination	A statement that adds substantially to, clarifies, explains, reveals, or enlightens – including their interpretation of data, conclusions, and results. Often in the middle of a conversation, these messages push conversation in a new direction by offering a new perspective, often forcing someone to think of someone in a new light.	@FireEMSChief There was probably a little leeway between 30 and 60. Also the breathalysers were reasonably inaccurate for this sort of thing.	336	24%
Opinion	The substantive idea that a person has about something or someone, which is based mainly on their personal feelings, beliefs, experiences or views.	agree w @ketaminh bad hypotension with verapamil I have good results with dilt @MDaware @RAGEpodcast @stemlyns	270	20%
Humor	Attempting to offer a funny or comical slant to a topic in discussion.	As everyone leaves for #smaccus, ketamine use plummets in EDs around the world...	165	12%
Reflection	Meditation or serious thought about one's character, actions, professional practice, and motives with purpose of understanding self or situation.	Sitting amongst the debris of Monday, picking up pieces of rubble & turning them over. My hands are grubby with start of week dust & decay.	31	2%
Networking	Interacting to meet professionally, exchange information, or develop contacts – especially to further one's career or social network.	.@PEMEMS @artangelo I'd be happy to look at what you sent, but I meant he should DM me too. I'd be happy to send him resources.	62	5%
Self-promotion	Publicizing one's own activities, including linking to one's own work if overt about one's role. If linking to own work but not explicit about author’s role, it is not self-promotion.	Excited to be publishing in the new @STEL_BMJ journal! Excellent review process - #MedEd / #Simulation researchers consider contributing.	23	2%
